# Supplementary figures and images for: Deciphering the Transcriptomic Complexity of Yak Skin Across Different Ages and Body Sites
Source: Int J Mol Sci. 2025 May 11;26(10):4601. doi: 10.3390/ijms26104601 (PMC12111109; doi:10.3390/ijms26104601)

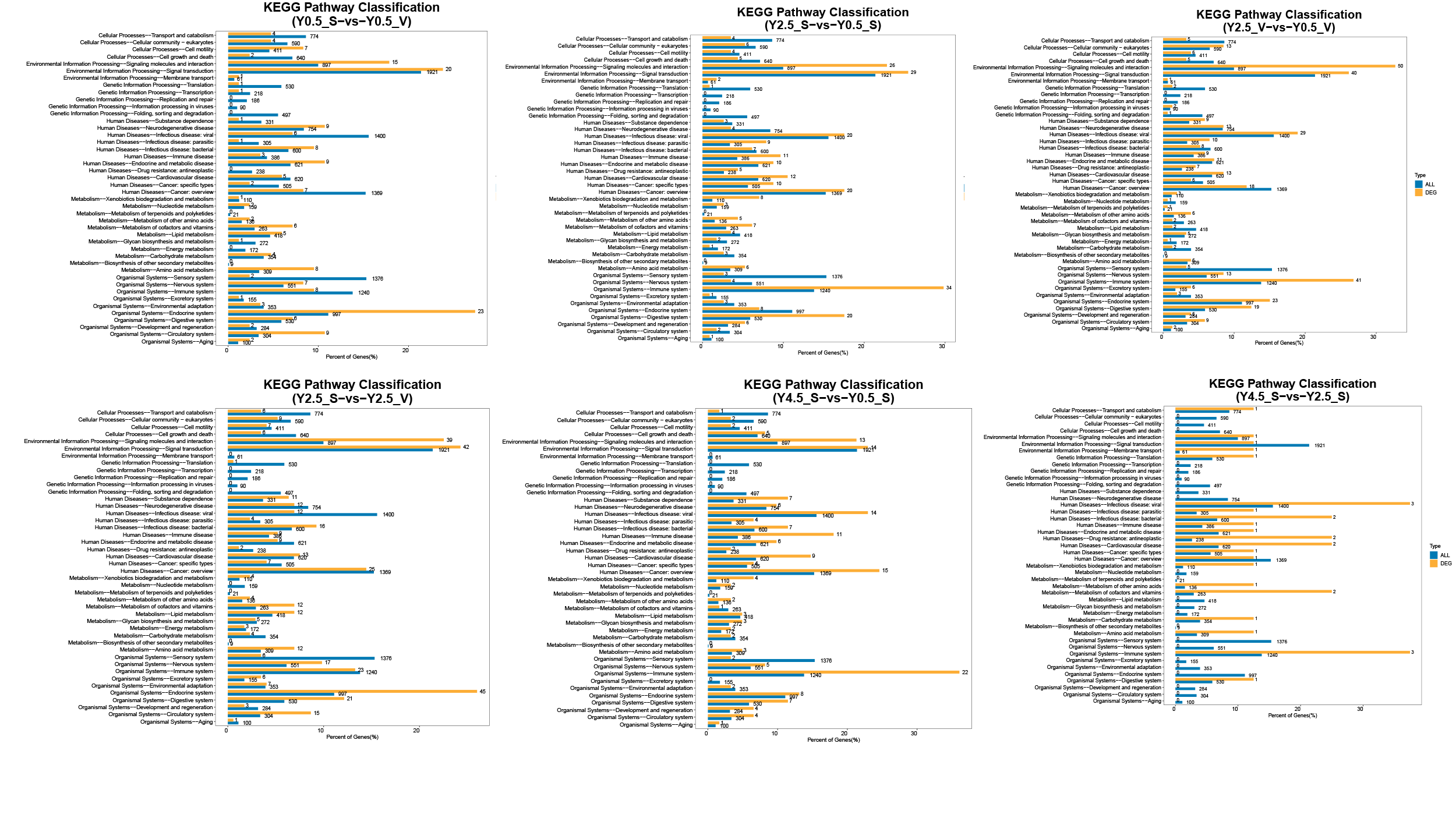

Supplement: Supplementary file 1 [file ijms-26-04601-s001.zip › Figure S1 KEGG pathway secondary classification of DEGs.tif]

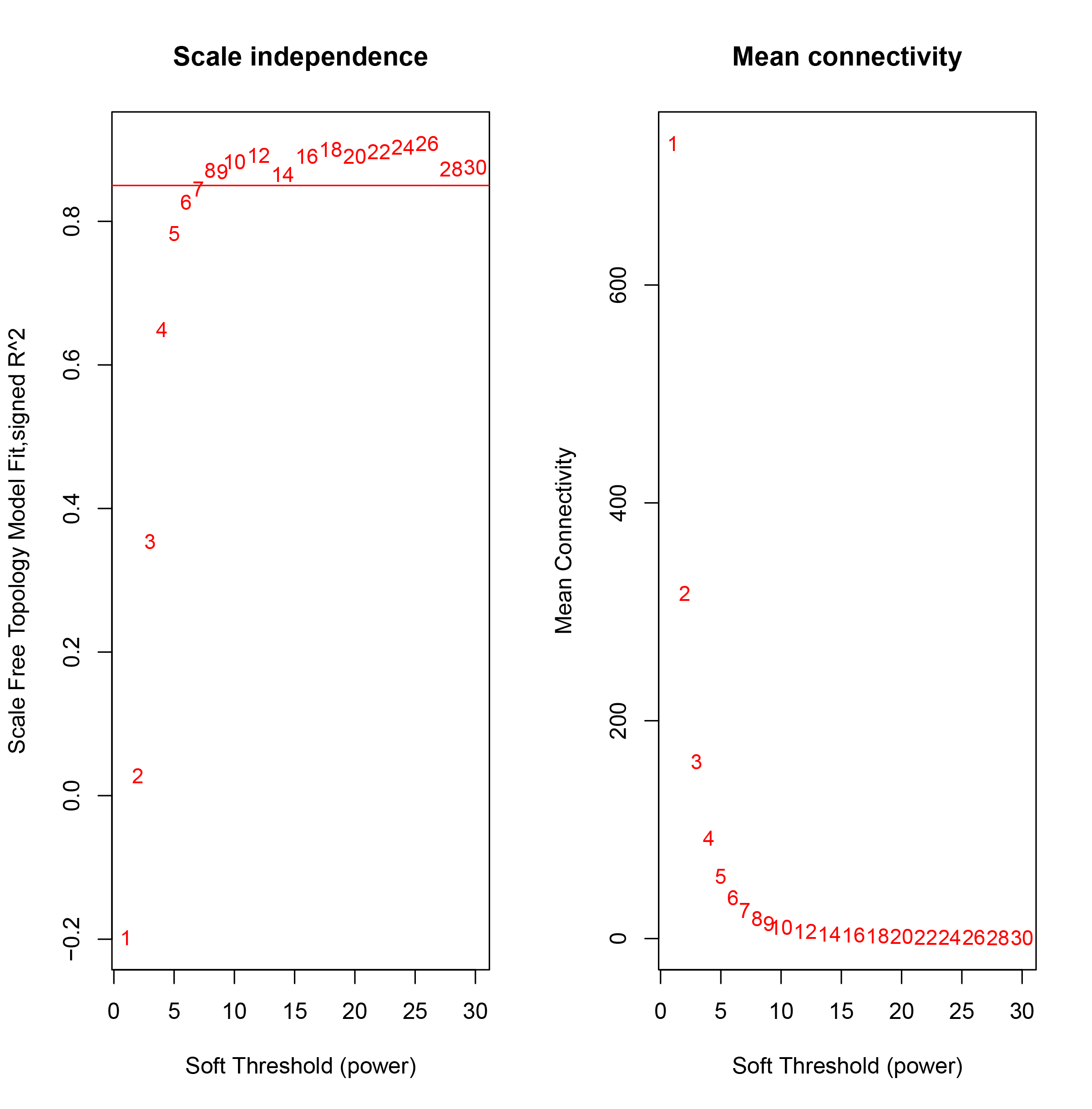

Supplement: Supplementary file 1 [file ijms-26-04601-s001.zip › Figure S2 Soft Threshold (power).tif]
